# Have adults lost their sense of play? An observational study of the social dynamics of physical (in)activity in German and Hawaiian leisure settings

**DOI:** 10.1186/s12889-016-3392-3

**Published:** 2016-08-02

**Authors:** Ansgar Thiel, Hendrik K. Thedinga, Samantha L. Thomas, Harald Barkhoff, Katrin E. Giel, Olesia Schweizer, Syra Thiel, Stephan Zipfel

**Affiliations:** 1 Institute of Sport Science, Eberhard Karls University of Tübingen, Tübingen, Germany; 2 Centre for Population Health Research, Faculty of Health and Social Development, Deakin University, Melbourne, Australia; 3 Kinesiology and Exercise Sciences, University of Hawai’i at Hilo, Hilo, HI USA; 4 Department of Psychosomatic Medicine and Psychotherapy, Medical University Hospital Tübingen, Tübingen, Germany

**Keywords:** Physical activity, Social dynamics, Group activity, Leisure settings, Observational study

## Abstract

**Background:**

Physical inactivity is one of the biggest health problems nowadays. Recent research shows that socio-cultural barriers to physical activity are mostly related to modern lifestyles. However, there is a lack of research on how social and group dynamics influence engagement in physical activity. Furthermore, there are few cross-cultural studies that have compared the social dynamics of (in)activity in different cultural settings. This paper therefore aims to analyse how social group dynamics influence physical activity and inactivity in informal social environments and whether physical activity is influenced by the socio-cultural settings.

**Methods:**

The paper presents the qualitative data collected within a covert participant observation study. Data was collected by keeping observational notes in order to record typical, regular patterns regarding physical (in)activity related behaviour of groups at an artificial open air swimming pool in Germany and a natural pond in Hawai’i. The data collection period was eight and a half months. Data was interpreted based on constant comparative analysis in order to identify most generative patterns in the field notes.

**Results:**

Group structures appear to play a significant role regarding the activity of the group members. In this study, we identified four key factors that influence group based physical activity: 1) Physical activity seems to be a group disturbing behaviour particularly in larger groups of adults; 2) Physical activity appears to be more functional and less joyful in adults than in children; 3) Group activity is influenced by (in)activity anchors, including ‘domestication’ of a group’s site, obesity, and controlling parents. 4) Physical activity is to a certain extent socially contagious, particularly with regard to playful activities.

**Conclusions:**

Successful promotion of physical activity should target the social structures of inactive individuals’ groups. In this regard, one of the main problems is that fun and wellbeing, as very important targets of public health strategies for the adult population, appear not to be compatible with physical activity. Developing strategies to reframe physical activity rather as ‘fun’ and less as functional may be one way to engage inactive individuals in physical activity in leisure settings.

## Background

The spread of noncommunicable diseases (NCDs) is increasing worldwide. In its new NCD report the World Health Organization (WHO) [1] states that by 2030 an average of 60 million people will die of lifestyle related diseases (including obesity, Type II Diabetes, and several cardiovascular illnesses). NCDs are caused by a complex range of individual, environmental, and commercial determinants [2–4]. The latest WHO report [1] states that on average 20 million NCD related deaths could be prevented by changes in lifestyle factors, and in particular, by increases in physical activity [5]. Understanding and targeting the reasons for inactivity is therefore an important factor in dealing with NCDs.

Apart from poor physical health, there are many socio-cultural barriers to physical activity which are predominantly related to modern lifestyles. These include lack of time, lack of opportunities, and lack of money for sports and leisure activities [6–9]. Others have also described how body dissatisfaction [6], and the stigma associated with being overweight or obese [10, 11] may prevent individuals from feeling able to engage in physical activity. Finally, a small amount of research suggests that dominant discourses about physical activity being a ‘counter’ for food consumption, may have shifted the ways in which individuals perceive, and engage in physical activity [12].

Furthermore, research has highlighted several factors influencing sedentary behaviour. Owen et al. [13] name - among others – age, socio-economic status, and psychological-behavioural factors as facilitators of and barriers to sedentary behaviour [13]. Importantly, Owen and colleagues also argue that especially environmental determinants such as “*behaviour settings*” [13] may play an important role. In a recent review, Gardner and colleagues [14] identify the social environment as one important factor for interventions in order to reduce sedentary behaviour.

In this regard, researchers have also studied how social networks may either encourage physical activity, or sedentary behaviour [15]. Some researchers have suggested that those who have low levels of physical activity have a much stronger influence on the physical activity behaviours of peers, as compared to those who are more physically active. This research shows that individuals imitate the behaviours of their ‘least fit’, rather than their ‘most fit’ friends [16]. While this and the aforementioned study have provided important insights about the social barriers of physical activity, much less is known about how social dynamics influence physical activity in smaller social groups. This includes how group behaviours relating to physical activity are impacted by cultural values and norms within different environments.

Socio-cultural factors play an important role in body type preference and body presentation [17–19]. From a symbolic interactionist perspective, cultural representations form individuals’ perceptions of how individual behaviour and appearance in a certain social contexts should be [20, 21]. Culture offers guidelines for typical situations: which social roles have to be played by whom and when, which sort of behaviour is allowed and how decisions are made [22]. The socially accepted body appearance and activity are therefore socially created and symbolized in stereotypical body shapes and somatic practices. In Germany, as has been similarly reported in many other western countries, obese bodies are perceived as both unattractive and unhealthy, while normal weight and athletic bodies are perceived as both healthy and the socially expected and desired norm [23, 24]. However, in other countries, the cultural norms around body weight are markedly different. In regions like the Pacific Islands, adolescents and parents desire a range of average-sized bodies that meet their culturally defined view of health [25], even though they do not desire obese bodies [25, 26].

The phenomenon that people follow cultural guidelines although they could in principal behave differently can be explained by the mechanisms of collective behaviour. Individuals have the tendency to bond with others who are similar to them [27]. In this regard, culture is a construct that has a very strong bonding force, offering culturally characteristic behavioural rules, beliefs and values, thus facilitating communication and relationship formation [22]. Sharing the same cultural beliefs can be seen as a process of building up ‘homophilic’ relationships, i.e. associating with similar others [16]. In this regard, the transfer of cultural values and norms to individual behaviour happens on the level of interaction, particularly within groups. Tajfel and Turner [28] define a social group as “*a collection of individuals who perceive themselves to be members of the same social category, share some emotional involvement in this common definition of themselves, and achieve some degree of social consensus about the evaluation of their group and of their membership in it*“[28]. The process of passing on norms, values, attitudes, and beliefs in groups can be regarded as a phenomenon of ‘social contagion’ whereby behaviour spreads between individuals in the form of imitations of actions, stimulated by another [29]. Several studies have provided proof for this imitative behaviour between individuals [27, 30, 31]. Social network research therefore shows that “*affect, attitudes, beliefs and behaviour can indeed spread through populations as if they were somehow infectious*” [29].

What is less clear from existing research, is how social and group dynamics influence engagement in ‘unsupervised’ physical activity in informal settings [32]. Furthermore, despite an acknowledgement that cultural factors may influence the way in which social groups interact in different environments, we know of very few cross-cultural studies that have compared the social dynamics of (in)activity in different cultural settings. Our observational study aimed to address this gap in knowledge by observing group behaviour in informal leisure settings which hold as little social, time-related, or factual activity barriers as possible in two culturally diverse regions: The Champagne Pond Pool on Big Island, Hawai’i, and the Entringen Pool in a small village near Tübingen, in the southwest of Germany. For the empirical study, a group was defined as a social collection of at least two individuals who are notably connected with each other, i.e. they arrived at and left the pool together, spent the majority of their time together, and shared one site for rest. The study was guided by three research questions:

1. How do social group dynamics influence physical activity and inactivity in informal social environments?
2. Which factors encourage or prevent individual group members from engaging in activity?
3. Do different socio-cultural settings influence physcial activity and inactivity in different ways?

## Methods

### Approach

Many studies about physical activity in leisure time are based on self-reports. Furthermore, there is a lack in research about “*unsupervised physical activity”* [32]*.* Correspondingly, McKenzie and van der Mars state that physical activity “*does not occur in a vacuum, but is place-based, and to fully understand it […], the context in which PA occurs must be considered*” [33]. In contrast to other methods, such as interviews, observations are able to provide such “contextually rich data” [33]. Therefore, we conducted a covert participant observation which allowed us to study naturally occurring physical activity and inactivity behaviour in groups.

This paper presents the qualitative data collected within a mixed method, covert participant observation study. This mixed method study consisted of two parallel ongoing data collections. The aim of the quantitative study component was to identify contrast groups of activity behaviour using complex statistical procedures. In this regard, we collected low-inference data about for example group size, estimated age or estimated body size of group members. In the following, we present data of the qualitative study component. Here we focused on (in)activity related group dynamics.

Following the suggestions by Sedlmeier and Renkewitz [34], we kept observational notes in order to record typical, regular patterns regarding the physical activity related behaviour of groups in the two study sites in Germany and Hawai’i.

### Research settings

Both research sites were selected because they are sized appropriately for an observation: they are not too large, so observers are easily able to keep groups within sight. Yet, both research sites still offer enough space in order to give sufficient opportunities for various physical activities as well as inactive and sedentary behaviour (sun-bathing and resting). Therefore, we had the opportunity to observe group dynamics in relation to these behaviours at these sites. The activity characteristics of the two research sites are detailed in Table 1. The first observation site was at a natural pond, the Champagne Pond Pool in Kapoho, Hawai’i, with an area to lie and sit which is made up of coarse-grained, black lava stones, and was approximately 50 m long. There is also a second area to sit and lie opposite the pool, which is about 300 square meters in size. The pool is about 200 m long and allows ball and tag games near the shore but also swimming and diving. The Champagne Pond Pool is publicly and freely accessible. However, many visitors travel to the pond by jeep or truck as access by foot is difficult. The second site was at an artificial open air pool, the Entringen open air pool, in the southwest of Germany. The open air pool area includes three separate pools: a large one (16 × 25 m) for swimmers, a medium pool for non-swimmers (12 × 12 m), and a small paddling pool for young children (6 × 6 m). It also has a very large lawn for sunbathing (approx. 100 m × 50 m). There is an equally sized playground lawn with a beach volleyball pitch and two very small football goals. The area also has a small playground for children, with table tennis tables. The Entringen open air pool is open to the public with a very small fee for visitors (three euros for adults/1,50 for children and students per visit).

**Table 1:** Activity characteristics of the research sites

Champagne Pond Pool and Entringen Pool
• Swimming (lap swimming as well as play)
• Diving activities (competitive and play)
• Aqua jogging (usually with swim noodle or inflatable mattress)
• Teasing one another and dunking each other under water, water splashing and water bombs (mostly children and adolescents)
• Treading through pool with long breaks at the edge of pool (cooling off in water when it is very sunny and hot)
• Ballgames in water/pool or on lawn (passing or shooting off friends)
• Wild, playful running, walking around (mostly young children)
• Slow/moderate walking around/playing with children by parents
• Playful jumping into pool or water
Entringen Pool
• Table-tennis, beach volleyball, football (mostly ball passing)
Champagne Pond Pool
• Snorkelling (usually while swimming slowly, sometimes supported by swim noodles)
• Stand up paddle boarding
• Canoeing
• Surfing and wakeboarding (outside the lava stone wall of pool)

For images of both research sites please click on the following link: http://www.ifs.uni-tuebingen.de/institut/arbeitsbereiche/sozial-und-gesundheitswissenschaften/forschung/observation/images.html

### Data collection and sample

In order to achieve observational “*data credibility*” [33] for the study, author (AT) developed an observer training protocol, and trained the other observers. The observers had to learn about the specifics of the planned observation and our research project at large. They also had to be instructed on *“ethical issues, the need for objectivity, maintaining confidentiality, and observer etiquette”* [33]. This was of particular importance since the observation was to be conducted covertly. To guarantee valid field-based assessments regarding BMI, body shape and age, the observers had to train with photographs and BMI models on the computer. In order to check the accuracy of body shape and age category assessments, we conducted a test where the observers had to assess 162 photographed people on the computer. The results are shown in Table 2.

**Table 2:** Results of rating

Sample n	162	
Images n excluded	5	(bad quality)
Sample n used for rating	157	
Mean Age	30,38	
Mean BMI	22,73	
Total ratings by all observers: 471		Error Rates Total:
Age Category Correct		96,60 %
Age Category Errors		16
Age Category Error Rate		3,40 %
Mean Deviation (in years)		2,83
Body Shape Category Correct		97,24 %
Body Shape Category Errors		13
Body Shape Category Error Rate		2,76 %
Mean Deviation (BMI)		1,38

Data was collected by author (AT) and author (ST) in Hawai’i, and author (HKT) and author (OS) in Germany. Field notes were made in German at both sites. Recorded notes were frequently discussed between observers. During the observations at the Entringen Pool, author (HKT) and author (OS) received continuous supervision by author (AT). All observers were from Germany. Author (HB) lives in Hawai’i and provided the team with cultural advice during data collection.

At each research site, the observers initially spent around one week in order to establish relevant categories for the coding. We developed initial ‘primary’ categories before recording started. During the data collection period, more ‘secondary’ observational categories were added. Primary and secondary observational categories can be found in Table 3.

**Table 3:** Observational categories

Primary observational categories	Explanation
Physical activity level of group	Observed physical activity behaviour of all members of a group. We recorded all observed aspects concerning physical activity and inactivity. Furthermore, we differentiated between ‘very active’/‘moderately active’/‘rather passive, sometimes in the water, with a maximum of two short active exceptions’/and ‘extremely passive’.
Research setting	Champagne Pond or Entringen Pool
Gender composition of group	Male only/female only/mixed
Combination of group concerning age	Concerning age, we differentiated between ‘children’/‘adolescents’/‘young adulthood’/‘middle adulthood’/and ‘seniors’.
Combination of group concerning body shape	Concerning body shape, we differentiated between ‘lean, normal-weight, and slightly overweight’/‘clearly overweight’/and ‘athletic’.
Group size	In this category, we differentiated between small groups including two or three members/middle size including four or five members/large groups including six or more.
Additional equipment	We observed whether groups had equipment such as chairs, sun loungers, barbecues, cooling boxes, sunshades, etc. or not.
Communication level within group	We observed the verbal communication of group members, e.g. loud or quiet. Moreover, we differentiated between frequent and lively verbal communication between most group members/occasional verbal communication/almost no verbal communication among group members.
Food and drinking behaviour	We recorded whether groups brought food and drinks to the sites.
Secondary observational categories	Explanation
Design of resting site	We observed, how groups organised their resting site, for example how they arranged their equipment, how they furnished their site etc.
Figurations	We observed in-/out-group constellations, age combinations at the site and group compositional changes.
Social contagion	In this context, we looked at the spread of activity within groups and between groups.
Area of rest and movement	We observed where members of groups spent the majority of their time resting and the other areas they moved to.
Promoting physical activity	We used this category for coding all factors which appeared to promote physical activity.
Hindering physical activity	We used this category for coding all factors which hindered physical activity.

Subsequently, one week (with daily visits of location) of explorative observation took place in order to give observers the opportunity to receive “*live field-based practice*” [33] before the recording started. The (recorded) observations at the Champagne Pond Pool took place on both week days and weekends from August 1^st^ 2012 to January 1^st^ 2013; and at the Entringen Pool from July 1^st^ to September 15^th^ 2014. Total numbers of days of observations was 156 (105 days in Hawai’i; 51 days in Germany). The difference in observational days between Hawai’i and Germany is firstly caused by different climate conditions, which in Hawai’i allowed us to collect data from August until January, while in Germany the bathing season only went on from June until early fall. Furthermore, the number of rainy days during the observation period was comparably high in Entringen, which additionally limited observation opportunities.

On the days when recording took place, the observers spent – depending on the weather – between one to four hours at the respective location, constantly moving around the site to ensure that they were not obviously seen as collecting data. Attending the research sites alone or in pairs was not unusual. Therefore, observers could attend the pool and the pond as a couple of visitors without drawing attention. In order to blend in, all observers wore leisurely outfits such as swimming trunks, tank tops, and flip flops. They also had to bring bath towels. Since many visitors brought a rucksack or a bag, observers could bring their observational diaries, notes, and pens with them in a rucksack. However, in order to remain unnoticed by other visitors, the observers often had to write down their observational notes outside of the observation areas. Data collection stopped either due to data saturation, weather conditions, lack of groups or time constraints.

### Data interpretation

Data interpretation was led by author (AT) and author (HKT). We started with a first data interpretation whilst data collection in order to extend observational categories. The main systematic data interpretation took place after data collection. The first steps in interpreting the qualitative field notes involved several readings and re-readings generating initial codes across the entire dataset. While first interpretation was based deductively on initial categories such as group size or gender, ongoing data analysis was inductive in order to find new factors and patterns in the field notes. An important aspect here was to focus on very frequent observations in the data. Differences between sites were discussed by the different observes. Author (AT), who took notes in Hawai’i, discussed such cases with author (HKT) and author (OS). Based on constant comparative analysis the observational data was subsequently organised by identifying recurrent basic themes and patterns in the observations. These ‘basic themes’ were then summarized by more abstract ‘organising themes’. Basic and organising themes were discussed with members of the research team, with consensus achieved about the main thematic clusters within the data. Based on the work on thematic networks by Attride-Stirling [35], these main-themes were used as so called ‘global themes’ which summarize all principal patterns of the organising themes and basic themes. In a final step, four global themes were defined to give an idea of the most generative patterns in the gathered data with regards to possible factors influencing physical activity.

## Results

**Fig. 1:**
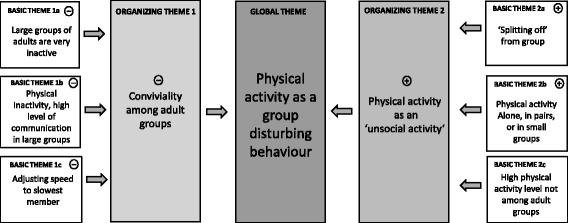
Overview of qualitative observations. Thematic Network One: Physical activity as a group disturbing behaviour

**Fig. 2:**
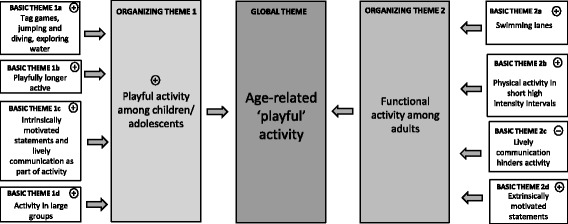
Overview of qualitative observations. Thematic Network Two: Age-related ‘playful’ activity

**Fig. 3:**
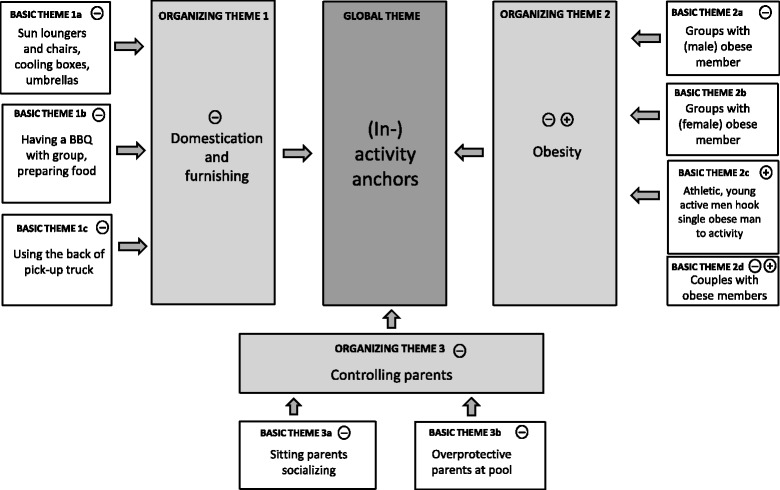
Overview of qualitative observations. Thematic Network Three: (In-) activity anchors

**Fig. 4:**
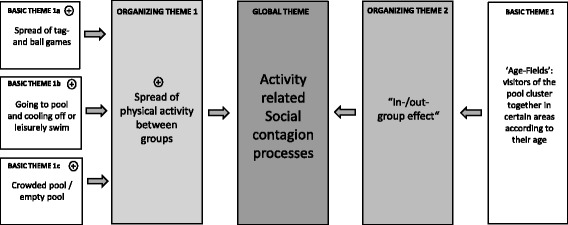
Overview of qualitative observations. Thematic Network Four: Activity-related social contagion processes

Four key themes emerged from the qualitative data: 1) Physical activity as a group disturbing behaviour; 2) Age-related ‘playful’ activities; 3) (In)activity anchors; 4) Activity related social contagion processes. The themes are presented in Figs. 1, 2, 3 and 4. Within these figures, the + signs relate to factors that encourage physical activity, and the – signs relate to factors that hinder physical activity. Throughout the result section, we include extracts from our observational notes to support the themes.

### Theme One: Physical activity as a group-disturbing behaviour

In both observational settings increased social interaction and communication was associated with more sedentary behaviour. For many adults observed in groups across both sites, the pools represented places of rest, dominated by sitting, talking, and eating, with large groups (≥4 people) particularly characterised by sedentary behaviour, and a high level of verbal communication. In Germany and Hawai’i, we observed that the more group members talked and communicated with each other, the less physically active they were. However, it was notable in both Hawaii and Germany that not only the more a given group of adults communicated the less physically active its members were during their stay, but also the larger a group was, the more physically inactive its individual members tended to be. Physical *in*activity became a specific group norm that helped to hold the group together, despite all activity opportunities the settings offered. In Hawai’i, this effect became even apparent in the spatial arrangement of the group:

Physically inactive groups tend to verbally communicate a lot. The groups position themselves almost as a united structure. On the beach: often on chairs, sitting next to each other and talking vividly. In the water: they often form closed groups, arranged in circles. They dwell in the water for hours without any discernible activity […] the mood in these groups seems to be very good and cheerful. […].

(Champagne Pond Pool)

Adults who came to the spaces by themselves were more engaged in physical activity than when they attended with their peers. For example, the researchers observed one woman, who attended the Entringen Pool over several days. When she attended the pool alone, the researchers observed that she engaged in much more physical activity, than she did when she attended with her friends. The larger the group of friends, the less active she became:

A woman (approx. late twenties, slightly overweight) and her physical activity behaviour could be observed on several days in two consecutive weeks. The following observations were thus subsequently recorded:

- (2×) **on her own**: 20–40 min. swimming of lanes on a moderate-high intensity [+10 min. of sunbathing]
- (3×) **together with one friend** (same age, female, sporty athletic): 2 × 15–20 min swimming of lanes on a moderate intensity [+ more than 30 min. of sunbathing]
- (1×) **together with two friends** (one friend above, the other: same age, female, overweight): 1 × 10–20 min swimming of lanes on a moderate intensity [+ very long sunbathing]
  - o (2×) **together with a group of five** (all approx. same age, male and female): hardly any swimming, maximum of 2–4 lanes, mostly lively discussion in group, eating, and sunbathing).

(Entringen Pool)

In both Hawai’i and Germany, physical activity was more likely to be undertaken with smaller groups of individuals (≤2–3 people). Individuals who planned to be vigorously active usually came to the pool or the beach on their own or with a friend/partner in order to swim. These individuals came to the pool with the specific purpose of exercising. They rarely stayed longer than the period of exercise, often leaving immediately after the activity had finished.

Very physically active individuals are mostly alone or in groups of two. The purpose of their visit is almost always connected to physical activity – mostly vigorous. Except for a towel and snorkel utensils they do not bring anything, and they hardly stay longer than the time in which they are physically active.

(Champagne Pond Pool)

If members of a larger group wanted to be physically active they, thus, usually ‘split off’ from the group, as if they did not want to disturb the rest of the group. This held particularly true for very active individuals, even when a group started being physically active together:

A group of three elderly (two women and one man) come together to the pool and start swimming lanes (moderately). After a while the man separates from the group and swims on his own (faster).

(Entringen Pool)

In both sites, if all members of a group were physically active together, we often could identify a strong influence of unspoken group rules on the group’s behaviour. One such unspoken rule appeared to be the decreasing of physical activity as demonstrated in the following observation of a group of three women in Entringen:

Three women swim their lanes on a moderate to high intensity separately in the pool. Especially one woman swims very fast. Then one woman joins the very fast woman, they start to talk a little, and swim their lanes together. The speed decreases instantly. When the third woman joins the group, the speed becomes even slower; they even stop at the edge of the pool in order to have a lively discussion. Only when the group dissolves, and the three swim separately, the speed does pick up again.

(Entringen Pool)

In a few cases, active group members tried to integrate the physically more inactive members by adjusting their activity level to the capacity of the ‘weakest’ member. From this perspective, the spread of inactivity (or less activity) ensured that the less active members of the group were not excluded from the activity. The following example shows that less active group members function in similar cases as ‘reverse pacemakers’:

A group of three middle-aged men go to the pool. Two of them (normal-weight) start swimming lanes on a moderate level while the third (overweight) stays passively at the edge of the pool. After two lanes the two swimmers come back to their friend and stay with him at the pool’s edge.

[They leave pool and come back later for a second swim]

This time they all swim a couple of lanes together, but in a considerably slower pace than before, adjusting their speed to the slowest. When the slow swimmer leaves the group and the pool, the two remaining pick up their ‘normal’ speed again.

(Entringen Pool)

We also could observe ritualistic customs associated with certain group behaviours:

Many groups set up their equipment ritualistically: first, they set up their seating arrangements, chairs and sun-loungers, then usually a period of rest follows.

(Champagne Pond Pool)

### Theme Two: Age-related ‘playful’ activity

In contrast to adult groups, conviviality in children and younger adolescent groups did not hinder physical activity at all. In contrast to adults, social communication and play held children’s groups together, and was the norm, even in very large groups of children:

For children and younger adolescents, the nature of conviviality is exactly the opposite as for adults. For them conviviality is almost always connected to being physically active. Conviviality is usually expressed by showing, imitating, playing together, teasing, or competing physically. Social interaction is established via physical activity and exercise.

(Champagne Pond Pool)

Interestingly, in our observed cases verbal communication was a motivating stimulus to physical activity in children. While in adults all physical activity, and especially vigorous exercise, lessened or stopped when the group members had lively discussions, verbal communication in groups of children/adolescents was an integral part of boisterous physical activity. Children and adolescents talked and even screamed notably loud while being active.

A group of three boys are playfully active (jumping, swimming) at the pool’s edge: one boy says, “You should try a somersault under water! That’s so cool under water!” After that the group further explores the medium water, “Look! How I can walk in the water!” A little while later the group is trying different jumps into the pool. Another boy loudly exclaims, “You have to try this jump!” jumps into pool, “that’s so much fun! What’s your favourite jump?” The group is physically active (on a lower intensity level) like this but for almost two hours. The group members are continually motivating themselves by similar comments during the entire activity period.

(Entringen Pool)

What one can immediately notice in this example is the intrinsically motivated and expressive nature of children when they are engaged in activity. Children expressed their enjoyment and fun attributing it directly to the activity at hand (jumping, diving, and experiencing the water). This coupling of intrinsically verbal motivation with ongoing playful activity may lead to a change in experiencing the duration in the sense that these children and adolescents simply seem to forget about the time they were spending engaging in the activity. In our example, the children’s cheering functions as a continuous activity motor and as a contagious stimulus for prolonged physical activity. Though the intensity of children’s activities were often only moderate, they were physically active notably longer than adult groups.

In contrast to children, activity stimulating verbal communication in adults was mostly referred to health or body shape and had an explicit extrinsic motivational content. Motivational statements were predominantly functional as adults often struggled to maintain long and/or playful activity periods. The following example is typical for functional physical activity periods in adults. Functional activities, like swimming lanes, are apparently not considered as something per se enjoyable but as a duty and task beneficial for one’s body and shape.

While a senior elderly woman is moderately swimming her lanes, a middle-aged woman arrives at the pool. The older woman obviously knows her, slows down and greets her thus, “So you managed to get here after all!?” The middle–aged woman stops and replies, “Yes, I was able to pull myself together in order to swim a couple of lanes.” The older woman jokingly declares while starting to swim again, “Very good, for that you get an A from me – a straight A!” Both women swam lanes for 20–30 min with a moderate intensity.

(Entringen Pool)

Except in children and young adolescents, boisterous playful activity was only observable in groups of young, mostly male adults who were active as part of partying. In these groups, casual verbal comments played a significant role to keep the activity running.

A group of young men is standing in the water (up to the hips), talking to each other and drinking beer. One of the group members suddenly caught a football and said to the others, “Come on, you lazy bones, don’t fall asleep”. The group plays for almost an hour, throwing and catching passes. The group members stimulate each other by competing playfully. Stimulating comments are for example: “You’re talent free” or “You’re as slow as a slug”. Although the comments lead to a continually increasing intensity of the game, the group members do not put away their beer bottles.

(Champagne Pond Pool)

### Theme Three: (In-)activity anchors

In both Hawai’i and Germany, we could identify ‘anchors’ which either ‘hooked’ group members to a permanent position and limited both their activity radius and intensity (*in*activity anchors) or ‘hooked’ them to an ongoing activity (*activity* anchors). There were three main (in)activity anchors. The first was the ‘domestication’ of a group’s site within the space. The second related to having predominantly obese members of a group. The third related to parents’ control of children’s behaviours.

The more a group ‘furnished’ their location at the pool with equipment such as chairs, coolers, or barbecues, the more inactive and passive its members tended to be. This was especially the case at the Champagne Pond Pool:

Many groups “domesticate and furnish” their site with pick-up trucks, tents, and barbecues. In these sites, visitors drink beer, eat, and are stuck to their chairs. Preparing food and eating is downright celebrated and has a high social function.

(Champagne Pond Pool)

In Germany, this ‘domestication’ and ‘furnishing’ was less frequent, the equipment was mostly limited to chairs and sun loungers, eating and drinking was more ‘functional’. Nonetheless, it seemed to hinder the activity of the groups significantly.

Obesity also functioned as a second inactivity anchor. Before we explain obesity as an inactivity anchor it is important to note that there was a difference in the way obese people ‘presented’ their body between the two research sites. In Hawai’i, obese people presented their body quite openly without any inhibitions, and frequented the pool at all times. However, in Germany, obese people more often hid their body with clothes and visited the pool especially in the morning or late afternoon when the pool was less crowded. Furthermore, obese people sat down in all areas at the Champagne Pond Pool, whereas they did more often sit down in the periphery at the Entringen Pool. However, despite these differences, obesity functioned in both settings as an inactivity anchor especially for obese men. Groups which incorporated male obese members were mostly physically inactive, independent of the weight of the other members.

In weight-mixed male groups, normal-weight members often imitate the activity behaviour of passive overweight members. The activity level here is very low (i.e. mostly sitting passively). This is already the case when there is only one overweight individual in the group who is very passive. This effect is even more discernible when the passive person is very obese.

(Champagne Pond Pool)

In the above described examples the minority, i.e. the passive overweight members, sat and chatted while the normal weight members did not express the desire of being physically active. Inactivity therefore ‘hooked’ the rest of the group to their position. The only exception for this phenomenon that could be observed was single young obese men in groups of predominantly very athletic, young, and active men. In these cases, the obese individuals did not have a limiting influence on the physical activity level within that group. This could be because the athletic and active groups were very committed to being active, and thus, functioned as activity anchors and ‘hooked’ the more passive minority to their activity. We observed this at both the German and Hawaiian sites. For example, the following observation showed that young men were engaged in activity with their peers if they were with a much more active male group:

Young obese men show the same level of activity as their peers if they are part of a young, very athletic, male group. Such groups are usually very active, throwing balls and doing short jumping in the water. In these cases, the young obese men play a very relevant role, particularly when the group plays cannonball landing.

(Champagne Pond Pool)

A minority of obese women in an otherwise male, normal weight group also did not have a limiting influence on the physical activity of the rest of the group. However, in contrast to the young obese men, the obese women themselves did not get ‘hooked’ by the activity of the majority. They were, therefore, only very rarely active during the activity periods of the normal weight group members. Instead, the active men simply separated from the passive, obese women. The following example illustrates this phenomenon:

Two obese women and four normal weight men are together at the beach. All of a sudden the four men simply separate from the two women and leave them behind in order to be active. The women stay back, talk, and prepare food. When the men come back, they do sit only for a while in order to eat. Yet, they quickly leave again in order to continue with their ball games.

(Champagne Pond Pool)

Obese women only had a limiting influence on the activity of connected others if they were in partnerships with normal weight men. Such couples rarely became active, and if so, the intensity of their activity was very low and did not interrupt their conversation.

Couples of obese women and normal weight men are usually inactive, taking a sunbath, talking or reading. They only rarely have a barbecue, the level of ‘domestication’ is rather low. Eating is mostly restricted to snacks, such as chips. In active periods, both partners are usually active together. The women often use tools, such as swim noodles, their normal weight partners swim beside them. As a rule, this activity is characterized by a continuously ongoing conversation.

(Champagne Pond Pool)

For obese men, however, a younger slim or normal weight female partner could function as an activity anchor. In these cases, the physical activity of the obese men generally seemed to be aimed at living up to the partner’s (supposed) expectations, like the following example illustrates:

A couple of young pensioners is swimming lanes. The husband (very overweight) takes a break at the edge of the pool and explains to another pool visitor: “40 lanes – ah yes – you know in order to stay in shape. But my wife does only 20.” He adds laughingly: “but she doesn’t need to!” (The wife seems younger and is significantly slimmer than her husband).

(Entringen Pool)

A third social anchoring mechanism was observed in sitting parents who came to the pool with their children.

Mothers are often sitting in groups together on the sunbathing lawn while the children play and romp around them. These mothers often focus on their conversation which seems to have an ‘inhibitory effect’ on the children’s activity. During the stay, the playing children come back to ‘the anchor’ at regular intervals as if they would like to attract their mother’s attention in order to ensure they have not forgotten them. In their activity breaks, the children sit down beside their mothers while the mothers usually continue with their conversation.

(Entringen Pool)

Moreover, parents and adults who accompanied their children to the child specific pool alone also had an activity hindering effect on their children if they were not active themselves.

Parents who accompany their children to the pool alone, usually simply sit down at the pool’s edge on a bench, passively observing. This quite often has the effect that the children’s radius of movement is highly limited to the proximity of the sitting parents.

(Entringen Pool)

In the first case the parental activity hindering effect seems to be caused by the lack of the received attention which limits the children’s activity. In stark contrast to this lack of parental attention, the limitation of activity in the second case seems to be rather caused by overprotection instead. Nonetheless, in both cases the children orientate their behaviour towards their parent(s), who have a significant anchoring influence on the children’s activity level and radius.

### Theme Four: Activity related social contagion processes

Physical activity can spread from one group to another without any apparent verbal communication or other conscious motivational stimuli. This was frequently observed both at the Entringen Pool and at the Champagne Pond Pool:

Although there are many children present at the pond today, there is only very little physical activity to be recorded. The children are playing quietly at the water or sitting besides adults, playing individual games. This state goes on for quite a while. Suddenly one group of children starts playing tag games in the water. The activity is very loud and boisterous. The other children stop playing their individual games and watch the first group very interestedly. A short time later a second group follows the first group and plays tag games themselves. After a short while a major part of the children are active simultaneously. This goes on until one group stops and goes back to the beach. Now the reverse effect seems to happen. Group after group stops their activity until there is almost no children activity in the water anymore.

(Champagne Pond Pool)

In this recorded example, the first group of children acts as the stimulus for the imitative actions of the other groups (recipients). Physical activity, here in the form of tag games, appears to spread between the groups. A very similar effect was observed and recorded on multiple occasions by the data-collectors at the Entringen Pool in Germany, only with the slight difference that it was a leisurely swim or going to the pool and cooling off that spread between groups. Therefore, the pool was sometimes empty for a longer period of time and then all of a sudden very crowded. Social contagious effects could also be caused by an ‘in-/out-group-effect’ i.e. that groups dissociate from each other based on certain criteria. At the Entringen Pool in Germany, for example, it became apparent that visitors sat down in certain areas of the sun-bathing lawn according to their age. We could identify an area for adolescents, one for young adults, and a third one for middle-aged adults, elderly, and families. We named these areas ‘age-fields’. Interestingly, it appears that most visitors automatically ‘cluster together’ in the ‘right’ age field. In other words, most visitor groups seem to accept this implicit social age-differentiation rule, and orientate themselves towards their peers.

## Discussion

This study aimed to investigate the impact of group based behaviours on the physical activity or inactivity of individuals in two settings in Germany and Hawai’i. In particular, we were interested in how social group dynamics influenced the physical activity of groups members; and specifically whether there were specific socio-cultural factors which contributed to physical (in)activity behaviours, and whether these differed between two different cultural settings. In doing so, we sought to address gaps in research literature relating to how group dynamics may impact on physical activity behaviours. This is important because it gives us a more nuanced understanding of the range of factors outside of individual level that may influence behaviours, and thus may be used to develop interventions aimed to increase physical activity particularly in sedentary groups.

Before discussing the results, it is important to highlight some limitations associated with this study. First, the observers in Hawai’i were German and may not have culturally appropriately interpreted the behaviours of a predominantly Polynesian population. To overcome this author (HB) was from Hawai’i and provided the team with cultural advice during the study. Second, this study takes a snapshot of activities during their time at the pools. As we did not talk to any of the individuals we observed, we cannot make any assumptions about their physical activity behaviours outside of the observational settings and the observation period. Since we did not talk to visitors, we can also not make any assumptions about the intended purpose or motivation for attending the pool or the pond. Yet, a possible explanation for some of our observed behaviours could be the motivation to visit the site. For example, if a family only intends to socialise at the pond rather than to go swimming, corresponding behaviour can be expected. Third, in order to remain unnoticed by the observed subjects, the observers often had to write down their observational notes outside of the observation areas. In these cases, the observations had to be memorized for a certain time before they could be documented. This holds particularly true for Hawai’i, where a significant part of the observations was done from the water. Although the observers where trained in memorizing their observations, observational errors therefore cannot be ruled out. Finally, visitors of the Champagne Pond had to either drive by truck, walk for 200 metres over stony terrain, or swim into the Pond. Access was therefore more difficult than in Germany.

Based on our original research questions, the findings from this study raise three points for discussion. Firstly, group structures seem to play a very significant role regarding the activity of the group members. The social environment clearly shapes an individual’s behavioural output [36]. This holds also true for physical activity. Our data shows that among adults, group size influenced the physical activity behaviours of individuals within the group. We found that the larger a group was, the less physically active they tended to be. However, this was not the case for young adults and children. In large adult groups verbal communication also seemed to be usually livelier than in small groups. We thus assume that in large socializing groups physical activity disturbs this verbal communication and this results in sedentary behaviour. In order to limit the members to sitting and chatting and to maintain ‘physical communication discipline’, physical *in*activity becomes a specific group norm. Against the background of Turner’s general assumptions that conformity with group norms plays a central role for holding a group together [37], physical *in*activity functions as a group stabilizing element, despite all activity opportunities the settings offers. Conviviality in large groups was therefore both in Germany and Hawai’i characterised by sitting together in groups, relaxing and verbal communication in the form of frequent discussions, but not by physical activity. If groups of adults wanted to become active, they had to either ‘split off’ or come to the setting in small groups.

We also observed that physical activity level is moderated by so called ‘social (in)activity anchors’, such as domestication, obesity, and, in children, sitting parents. In this regard, mechanisms of social contagion are observable, such as the unconscious mimicry of the behaviour of significant others [30] or group dynamics driven by in-/out-group-constructions [28]. The phenomenon that even a small minority of a group can have a very powerful influence on the group’s behaviour might be explained by the findings of Xie et al. [38] who demonstrated that minorities can change the opinion of the whole group rapidly if they are larger than 10 % and, importantly, are very committed and dominant. We could observe – what Luszczynska et al. [31] and Dijksterhuis et al. [30] found in previous studies – that individuals imitate the behaviours of their peers automatically by unconscious mimicry in order to follow such unspoken rules. The data showed that this was especially the case for imitating physically inactive individuals. This observation is similar to other studies [16] that show that people tend to imitate the behaviour of their least fit, respectively less active friends. This may be a ‘courtesy effect’ whereby individual members of groups must not show their physical superiority but instead have to behave in such a way that the physically least fit member is not discriminated. This could particularly hold true for Hawai’i. The idea that group members must not be disadvantaged or embarrassed in public is an integral part of *Ohana*, a Hawaiian cultural ideal, that means family in a broader sense, including near friends. The idea of *Ohana* expects group members to build up strong relations with the others, to cooperate and help each other. In the context of these findings, an important first strategy to discourage inactivity anchors may be to make people aware of their activity hindering influence. This holds particularly true for inactive or overprotective parents who should be encouraged to play actively with their children instead of just watching them.

Secondly, there is a difference in the way adults and children are physically active. While adults had very structured forms of physical activity, children were more spontaneous and played and ‘celebrated’ physical activity in form of playful, boisterous group activity. A possible explanation might therefore be that children perceive physical activity as something that is separated from other activities by a specific, self-referential use of rules, spaces, and time structures [39]. The coupling of intrinsically verbal motivation with ongoing playful activity may lead to a change in experiencing the duration in the sense that these children and adolescents simply forget about time. This nullification of every-day life time structures is an important aspect of children’s games [40]. The observation that groups of children who play together do not seem to fatigue and are physically active longer goes in line with the findings by Scarapicchia and colleagues [41] who suggest that intrinsic motivation seems to spread and has a beneficial outcome on willingness to be physically active for longer. This observation might also explain why even large children groups were physically active in contrast to large adult groups.

Among adult groups, physical activity tended therefore to be an unsocial and functional activity. In other words, adults seem to have lost their sense of joyful, physically active play. Physical activity in adulthood appears to have rather functional purposes and is limited to shorter intense intervals, even in leisure settings. This goes in line with several recent studies which have suggested that adults (starting already at college age) increasingly perceive physical activity in general and especially exercise mostly as a lonely individual activity that is mainly driven by extrinsic motivations, such as health and attractiveness [42]. This represents a challenge for public health and health promotional specialists, particularly given that physical activity which is embedded in a social network is one of the most beneficial health factors [43]. Reframing the way in which adults think about physical activity – as a fun rather than function activity – may be a strategy for health promotion and social marketing initiatives which aim to stimulate activity both in adult and child groups [12].

A potential strategy could be to use fun and spontaneous activities for people of all ages and body-weights in order to engage them in more activity at leisure settings. An example could be ‘discovery paths’ in leisure settings which invite all visitors to actively explore the water, the beach, and the area together in groups by walking, climbing, and jumping. A further example would be game activities in groups such as ball games. Correspondingly, Kilpatrick and colleagues point out that motives for joyful play activities such as team sports “*are more desirable than those for exercise and may facilitate improved adherence to physical activity*” [42]. Our findings underscore the notion that in order to fully understand the complexity of causes which underlie physical inactivity one has to take an individual’s immediate social environment into account, in other words, the group with which an individual spends his or her free-time. Consequently, this study supports the view put forward by Fowler and Christakis [44] that due to “*our embeddedness in social networks […] group level interventions may be more successful and more efficient than individual interventions*” [44]. Specifically our findings concerning socially contagious physical activity between groups underscore this perspective. Socially contagious activity could be facilitated by providing stimuli. For instance, employees of an open air pool may encourage or ask one or two groups to start an activity as a stimulus for other groups.

Finally, although we expected that cultural settings would significantly influence physical activity behaviours in different groups across the two sites, we found only few marked differences in influencing social factors between Germany or Hawai’i. The most evident culture-related behavioural difference was the way in which individual obese people presented their body and occupied spaces. Yet, this did not directly impact their physical activity. One explanation for the lack of differences in the two cultural settings might be that we did not employ natives as observers in Hawai’i, and that the observers therefore lacked a complex understanding of certain socio-cultural mechanisms.

## Conclusion

In order to tackle physical inactivity it may be vital to target the social environment of inactive people, i.e. the structures of the groups to which inactive individuals assign themselves. In this context, one of the main problems will be the fact that for adults fun and wellbeing appear to be not an essential part of their physical activity. Yet, these aspects should be important targets of public health strategies for the adult population. Therefore, developing strategies to reframe physical activity as ‘fun’ and to employ different types of activities based around play rather than structured and functional activities, may be one way to engage inactive individuals in physical activity in leisure settings. More observational studies investigating physical activity behaviour are needed. In order to increase the representativeness of the research, these studies could be conducted in other leisure settings such as recreational parks. Furthermore, we recommend that future research may investigate our themes more closely in experimental approaches.
